# Editorial: One Health Approach in Zoonosis: strategies to control, diagnose and treat neglected diseases

**DOI:** 10.3389/fcimb.2023.1227865

**Published:** 2023-06-08

**Authors:** Patricia Flavia Quaresma, Erica S. Martins-Duarte, Lia Carolina Soares Medeiros

**Affiliations:** ^1^ Laboratório de Protozoologia, Departamento de Microbiologia, Imunologia e Parasitologia, Universidade Federal de Santa Catarina, Florianópolis, Brazil; ^2^ Departamento de Parasitologia, Universidade Federal de Minas Gerais, Belo Horizonte, Brazil; ^3^ Laboratório de Biologia Celular, Instituto Carlos Chagas, Fundação Oswaldo Cruz, Curitiba, Brazil

**Keywords:** One Health, parasitic infection, zoonosis, diagnosis, drug development

The increase and dispersion of zoonotic diseases, which include globally disseminated infectious agents shared between animals and humans, has become one of the most alarming threats to human health. Effective prevention and control of zoonotic diseases require a One Health approach that involves collaboration across sectors responsible for human health, animal health (domestic and wildlife), and the environment (Ghai et al., 2022). A faster and more accurate diagnostic approach clearly improves the prevention and control of zoonotic diseases. In addition, the development of better and safer drugs to treat zoonotic diseases is also urgent to achieve a reduction in the number of cases.

Due to the increased awareness of the different clinical manifestations caused by parasitic diseases, their diagnosis and treatment have undergone major changes. Newly developed diagnostic techniques allow faster and more accurate diagnosis of parasitosis caused by metazoans and protozoa. Furthermore, the increase in knowledge of parasite biology in recent years and the improvement of drug screening tools have led to the discovery and validation of new potential drug targets and molecules with antiparasitic activity. It is interesting to emphasize that diagnostic and treatment practices must be directed not only to infected humans but to other vertebrate hosts (reservoirs) involved in the transmission of zoonotic diseases. This approach fits within the concept of One Health, defined as “the collaborative effort of multiple disciplines to obtain optimal health for people, animals, and our environment.”

The ability to provide timely, accurate, and reliable diagnostic testing to detect and characterize the pathogen within laboratory networks (Belay et al., 2017) is central to any effective zoonotic disease prevention and control program. The sharing of best practices on diagnosis of zoonoses and the further refinement of new, cheaper, multispecies tests which can be interpreted by minimally trained individuals could contribute to a greater level of intersectoral integration, control, and elimination of zoonoses (Bidaisee and Macpherson 2014). A good example of the need for fast, easy to perform, point of care, and accurate diagnostic methods are the immunochromatographic tests used to diagnose human and canine Visceral Leishmaniasis (VL). Considering that the disease has an insidious evolution in humans, and can lead to death if not treated, an accurate diagnosis becomes even more important. In dogs, which are considered reservoirs of the parasite that causes VL, if the infection is not quickly detected so that treatment or euthanasia measures are applied, this infected animal will remain in the endemic area for a long time, contributing to the increase in the incidence of insect vectors infected (**Figure 1**).

**Figure 1:**
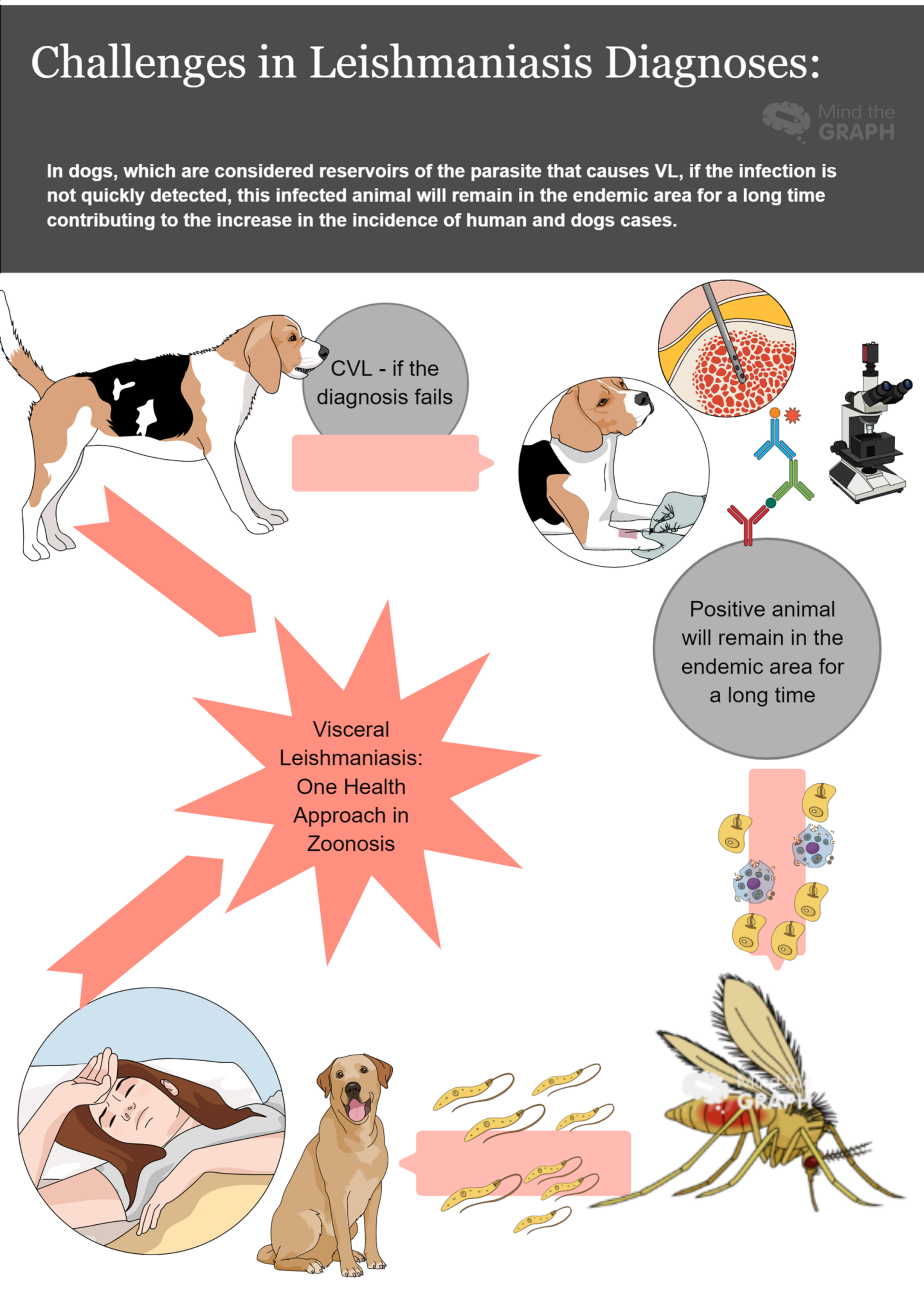
An example of the need for fast, easy to perform, point of care and accurate diagnostic methods: canine visceral leishmaniasis (CVL) must be diagnosed preliminarily to prevent a large number of insect vectors from becoming infected and transmitting the parasite to other vertebrate hosts.

In the present Research Topic, we gather six studies led by independent scientists that bring new insights and advances to the One Health approach in zoonosis research. In the diseases caused by an ectoparasite, Amanzougaghene et al. analyzed head and body lice collected from Mbuti pygmies and revealed the presence of two mitochondrial clades. Moreover, sixteen haplotypes were found in 47 samples of which thirteen were novel haplotypes, indicating an unusually high genetic diversity that closely mirrors the diversity of their hosts. One of the most interesting findings of the study was the report for the first time of the presence of the DNA of *Rickettsia felis* in head and body lice raising the question whether the *Pediculus* lice can indeed transmit this emerging zoonotic bacterium to their human hosts. Chienwichai et al. identified potential biomarkers of early Mekong schistosomiasis using an untargeted metabolomics approach. Three compounds were identified as potential biomarkers at the early stage of the disease: heptadecanoyl ethanolamide, picrotin, and theophylline. Goff et al. tested Raman spectroscopy as a diagnostic approach for Lyme disease patients caused by *Borreliella burgdorferi*, since the two-tiered serology, the only approved diagnostic test in the United States, include poor sensitivity, background seropositivity, and cross-reactivity. Liu et al. presented a comparative proteomics analysis of *Schistosoma japonicum* developed in different *Oncomelania* snails as intermediate hosts and helps to improve our understanding of the growth and developmental mechanisms of this parasite. Morais et al. characterized the activity of a new metallo copper-cinchonine complex (CinCu) against the asexual and sexual blood stages of *Plasmodium*, the protozoan responsible for causing malaria. CinCu significantly reduced the parasitemia *in vitro* and *in vivo* in a vertebrate model of infection, and suppressed the formation of oocysts in the invertebrate host, showing a promising activity as a future parasite-blocking transmission agent. Important to point out that the number of zoonotic malaria cases is increasing and can be considered an emergent disease (Lempang et al., 2022). In the study of Yang et al. RNA interference was used to investigate the impact of signal peptidase complex (SPC) components on the growth and development of *Schistosoma japonicum*. The data demonstrated that SPC is essential of *S. japonicum* survival and therefore provides a promising anti-schistosomiasis drug target.

In summary, the rise and spread of zoonotic diseases pose a significant threat to human health, imposing the need of a collaborative One Health approach. Improved diagnostic techniques have enabled faster and more accurate detection of parasitic diseases, leading to advancements in treatment and drug discovery. It is crucial to focus on diagnosing and treating infected humans as well as other vertebrate hosts involved in disease transmission cycle, aligning with the concept of One Health. This Research Topic presented studies that shed light on various aspects of zoonotic diseases, including genetic diversity in lice, biomarkers for early schistosomiasis detection, Raman spectroscopy for Lyme disease diagnosis, proteomics analysis of *Schistosoma japonicum*, the activity of a new metallo copper-cinchonine complex against malaria, and the impact of signal peptidase complex on the growth and development of *S. japonicum*. These advancements bring us closer to effectively combating zoonotic diseases and safeguarding human and animal health within a holistic framework.

## Author contributions

All authors have read, reviewed and approved the final text and contributed equally to this editorial.
